# Cardiotoxicity among socioeconomically marginalized breast cancer patients

**DOI:** 10.1007/s10549-022-06695-0

**Published:** 2022-08-15

**Authors:** Yan Lu, Aaron W. Gehr, Ifedioranma Anikpo, Rachel J. Meadows, Kevin J. Craten, Kalyani Narra, Anuradha Lingam, Sandeep Kamath, Bhavna Tanna, Bassam Ghabach, Rohit P. Ojha

**Affiliations:** 1 Center for Epidemiology and Healthcare Delivery Research, JPS Health Network, 1500 South Main Street, Fort Worth, TX 76104 USA; 2 Department of Public Health Sciences, University of Miami Miller School of Medicine, Miami, FL USA; 3 Oncology and Infusion Center, JPS Health Network, Fort Worth, TX USA; 4 Department of Internal Medicine, TCU School of Medicine, Fort Worth, TX USA; 5 Department of Cardiology, JPS Health Network, Fort Worth, TX USA; 6 Department of Family Medicine, JPS Health Network, Fort Worth, TX USA

**Keywords:** Breast cancer, Cardiotoxicity, Trastuzumab, Anthracycline, Safety-net

## Abstract

**Purpose:**

Evidence of cardiotoxicity risk related to anthracycline or trastuzumab exposure is largely derived from breast cancer cohorts that under-represent socioeconomically marginalized women, who may be at increased risk of cardiotoxicity because of high prevalence of cardiovascular disease risk factors. Therefore, we aimed to estimate cardiotoxicity risk among socioeconomically marginalized breast cancer patients treated with anthracyclines or trastuzumab and describe clinical consequences of cardiotoxicity.

**Methods:**

We linked electronic health records with institutional registry data from a Comprehensive Community Cancer Program within a safety-net health system. Eligible patients were adult females, diagnosed with first primary invasive breast cancer between 2013 and 2017, and initiated anthracyclines or trastuzumab as part of first-line therapy. We estimated cumulative incidence (risk) of cardiotoxicity with corresponding 95% confidence limits (CL) using the Aalen-Johansen estimator with death as competing risk.

**Results:**

Our study population comprised 169 women with breast cancer (103 initiated anthracyclines and 66 initiated trastuzumab). Cumulative incidence of cardiotoxicity was 21% (95% CL: 12%, 32%) at one year and 25% (95% CL: 15%, 35%) at three years among women who initiated trastuzumab, whereas cumulative incidence was 3.9% (95% CL: 1.3%, 8.9%) at one year and 5.9% (95% CL: 2.4%, 12%) at three years among women who initiated anthracyclines. More than half of patients with cardiotoxicity experienced interruption of cancer treatment.

**Conclusion:**

Our findings suggest high risk of cardiotoxicity among socioeconomically marginalized breast cancer patients after initiation of anthracyclines or trastuzumab. Strategies are needed for optimizing cancer treatment effectiveness while minimizing cardiotoxicity in this population.

**Supplementary Information:**

The online version contains supplementary material available at 10.1007/s10549-022-06695-0.

## Introduction

Anthracyclines and trastuzumab contribute to improved survival among women with breast cancer, but these drugs have well-documented adverse effects such as cardiotoxicity [1–6]. Cardiotoxicity following cancer treatment is defined as symptomatic or asymptomatic cardiovascular dysfunction. Symptomatic cardiovascular dysfunction is characterized by cardiomyopathy or congestive heart failure and asymptomatic cardiovascular dysfunction is characterized by decreased left ventricular ejection fraction (LVEF) without apparent symptoms.[2] The risk of cardiotoxicity ranges between 2 and 48% [3, 4, 7, 8] for breast cancer patients treated with anthracyclines or trastuzumab, but these estimates are based on populations that are over-represented in research [9]. Limited evidence is available about cardiotoxicity risk in populations that are under-represented in research such as socioeconomically marginalized populations [9, 10], which includes individuals with low-income, no health insurance, or inadequate health insurance [11]. The need for evidence about cardiotoxicity in socioeconomically marginalized populations is recognized by the National Cancer Institute [12].

The estimated risks of cardiotoxicity related to anthracyclines and trastuzumab reported in prior studies may not apply to socioeconomically marginalized breast cancer patients (i.e., lack of generalizability [13–15]). Socioeconomically marginalized breast cancer patients have a high prevalence of risk factors for cardiac dysfunction such as obesity and tobacco use [16], which may exacerbate cardiotoxicity risk [2, 17, 18]. Estimates of cardiotoxicity risk in this population could inform decisions related to addressing unmet needs for cardiotoxicity prevention, detection, and management. Therefore, we aimed to estimate cardiotoxicity risk among socioeconomically marginalized breast cancer patients treated with anthracyclines or trastuzumab and describe the clinical consequences of cardiotoxicity.

## Methods

### Study population

Our study population was derived from the JPS Health Network Oncology and Infusion Center, which is a Comprehensive Community Cancer Program in an urban safety-net health system [11]. The JPS Oncology and Infusion Center is the primary source of cancer care for socioeconomically marginalized populations in Tarrant County, TX. We linked institutional cancer registry data with electronic health records (EHR) to develop the study cohort. Eligible patients were women aged ≥ 18 years, diagnosed with first primary invasive breast cancer between 2013 and 2017, received at least part of their first course treatment at the Oncology and Infusion Center, and were either human epidermal growth factor receptor 2 (HER2)-positive cases that initiated trastuzumab or HER2-negative cases that initiated anthracyclines. We excluded patients with a history of heart failure or cardiomyopathy before trastuzumab or anthracycline initiation, or patients who had a baseline LVEF value < 55%. This study was approved by the North Texas Regional Institutional Review Board (IRB# 2017–109).

### Variables

Our outcome of interest was cardiotoxicity, defined as heart failure or cardiomyopathy using the International Classification of Diseases 9th (ICD-9) or 10th Edition (ICD-10) diagnosis codes (*Supplementary Materials, Appendix A, Supplementary Table S1*) that were confirmed by supporting documentation in EHR, or cardiac dysfunction indicated by drop of LVEF ≥ 10% from baseline to < 55%[2] after anthracycline or trastuzumab initiation. LVEF measures were obtained through echocardiogram, where we used the mid-point value if LVEF was reported as a range, or multigated acquisition (MUGA) scan. In addition, we extracted age at cancer diagnosis, race/ethnicity (non-Hispanic White, non-Hispanic Black, Hispanic, or non-Hispanic other), insurance status at diagnosis (uninsured with or without hospital-based medical assistance program for low-income qualifiers, Medicaid, or non-Medicaid insurance), marital status (single/unmarried, married, or divorced/separated/widowed), body mass index (BMI; < 25, 25 ≤ BMI < 30, or ≥ 30), tobacco use (never used, current use, or former use), alcohol use (never used, current use, or former use), hypertension at baseline (yes or no), diabetes at baseline (yes or no), American Joint Committee on Cancer (AJCC) stage (I, II, III, or IV) [19], grade (well differentiated/differentiated, moderately/moderately well differentiated, or poorly differentiated), and left-sided radiation therapy (yes or no).

### Data analysis

We estimated the overall and subgroup-specific (age and left-sided radiation therapy) cumulative incidence (i.e., risk) of cardiotoxicity with corresponding 95% confidence limits (CL) using the Aalen-Johansen estimator[20–22] to account for death as a competing event. Patients contributed time to the cohort from initiation of trastuzumab or anthracycline therapy to cardiotoxicity diagnosis (event of interest), death (competing event), loss to follow-up[23] (12 months after the last encounter with our health system), or end of study (December 31, 2020), whichever occurred first. We initially intended to dichotomize subgroups at age 60 years for comparability with prior studies, but the younger age distribution of our study population resulted in sparse data with this dichotomy. Consequently, we dichotomized subgroups at age 50 years to approximate pre- and post-menopausal status. In addition, left-sided breast cancer may be treated with radiation therapy, which may increase cardiotoxicity risk because of radiation exposure to the heart [2]. We thus included a subgroup analysis by left-sided radiation therapy. We did not adjust for covariates in our analyses considering covariate adjustment is contraindicated for descriptive studies and may mislead interpretation [24, 25]. All analyses were performed using Stata 16 (StataCorp, College Station, TX).

For clinical consequences of cardiotoxicity, we described impact on trastuzumab or anthracycline therapy completion, cardiotoxicity management, and cardiotoxicity outcome. Impact on treatment completion were categorized as cardiotoxicity occurred after treatment completed, completed treatment without interruption, treatment interrupted but resumed and completed, or treatment interrupted and did not complete. Cardiotoxicity management was categorized as treated with new medication or new doses of existing medication, continued with medications for existing cardiovascular disease, or observed/not treated with medications. Cardiotoxicity outcome was categorized as reversed or partially reversed (LVEF reversed to within 5% of baseline, improved by ≥ 10% but remained > 5% below baseline [26], or had clinical documentation of improved symptoms for patients without follow-up LVEF measurement) or not reversed (LVEF improved by < 10% and remained > 5% below baseline[26] or had no clinical documentation of improved symptoms for patients without follow-up LVEF measurement).

### Sensitivity analysis

The definition of cardiotoxicity varies across studies, which made risk estimates incomparable [27, 28]. Our primary analysis included the common definition of cardiotoxicity used by the American Society of Clinical Oncology [2]. Nevertheless, a stricter definition of cardiotoxicity is sometimes used. Therefore, we repeated our analytic approach using cardiotoxicity defined as heart failure or cardiomyopathy using diagnosis codes that were confirmed by supporting documentation in EHR, or drop of LVEF > 15% from baseline to < 40% after trastuzumab or anthracycline initiation [7].

## Results

**Fig. 1:**
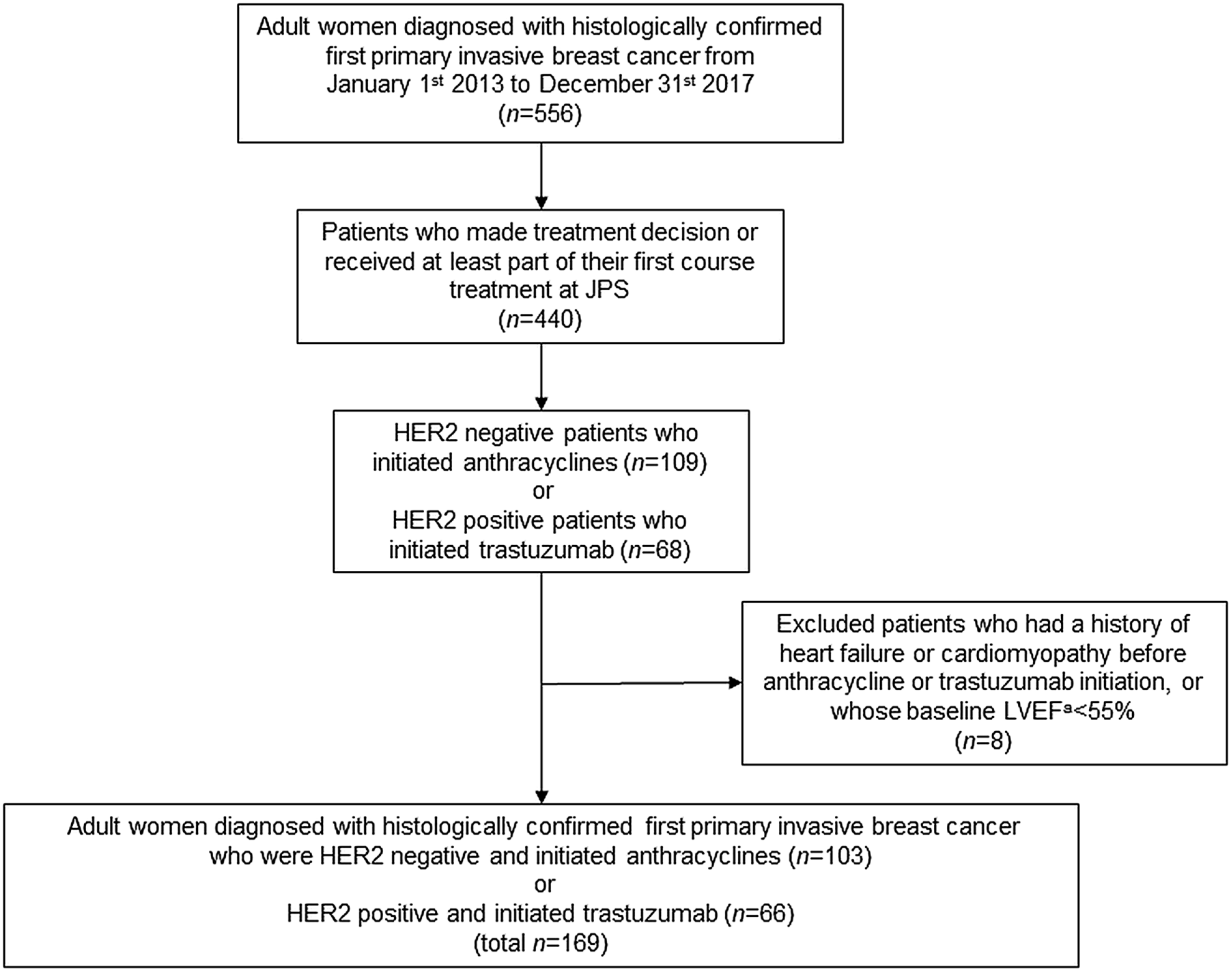
Selection of socioeconomically marginalized women diagnosed with first primary invasive breast cancer who initiated trastuzumab or anthracyclines. ^a^LVEF: Left ventricular ejection fraction

Our study population comprised 169 women with breast cancer (66 initiated trastuzumab and 103 initiated anthracyclines; Fig. 1), for whom the median age was 51 years (interquartile range: 44–58). Most women were racial/ethnic minorities (75%) and uninsured (57%). Among women who were uninsured, 46% were enrolled in a hospital-based medical assistance program for low-income qualifiers. Most women in our study population (56%) were diagnosed with stage I or II breast cancer. Table 1 summarizes characteristics of our study population by treatment group.

**Table 1:** Characteristics of socioeconomically marginalized women diagnosed with first primary invasive breast cancer who initiated trastuzumab or anthracyclines

	HER2-positive and initiated trastuzumab (*n* = 66) *n* (%)	HER2-negative and initiated anthracyclines (*n* = 103) *n* (%)
*Age (years)*		
Median (Interquartile range)	50 (43–58)	52 (45–58)
18–50	34 (52)	48 (47)
51 and older	32 (48)	55 (53)
*Race/Ethnicity*		
Non-Hispanic White	18 (27)	24 (23)
Non-Hispanic Black	21 (32)	37 (36)
Hispanic	18 (27)	31 (30)
Non-Hispanic other	9 (14)	11 (11)
**Insurance**		
*Uninsured*		
Without hospital-based medical assistance program	7 (11)	12 (12)
With hospital-based medical assistance program	26 (39)	51 (50)
Medicaid	10 (15)	8 (7.8)
Non-medicaid insurance	23 (35)	30 (29)
Missing	0 (0)	2 (1.9)
*Marital status*		
Single/Unmarried	24 (36)	40 (39)
Married	25 (38)	37 (36)
Divorced/Separated/Widowed	17 (26)	24 (23)
Missing	0 (0)	2 (1.9)
*BMI*		
BMI < 25	12 (18)	9 (8.7)
25 ≤ BMI < 30	12 (18)	31 (30)
BMI ≥ 30	42 (64)	62 (60)
Missing	0 (0)	1 (0.97)
*Tobacco use*		
Never used	42 (64)	62 (60)
Current user	11 (17)	25 (24)
Former user	13 (20)	15 (15)
Missing	0 (0)	1 (0.97)
*Alcohol use*		
Never used	56 (85)	87 (84)
Current user	9 (14)	13 (13)
Former user	0 (0)	2 (1.9)
Missing	1 (1.5)	1 (0.97)
*Hypertension at baseline*		
Yes	32 (52)	46 (45)
No	34 (48)	57 (55)
*Diabetes at baseline*		
Yes	15 (23)	26 (25)
No	51 (77)	77 (75)
*AJCC stage*		
Stage I	10 (15)	9 (8.7)
Stage II	32 (48)	43 (42)
Stage III	17 (26)	39 (38)
Stage IV	7 (11)	12 (12)
*Grade*		
Well differentiated/Differentiated	2 (3.0)	7 (6.8)
Moderately/Moderately well differentiated	25 (38)	34 (33)
Poorly differentiated	37 (56)	58 (56)
Unknown	2 (3.0)	4 (3.9)
*Left-sided radiation therapy*		
Yes	18 (27)	36 (35)
No	48 (73)	67 (65)

**Fig. 2:**
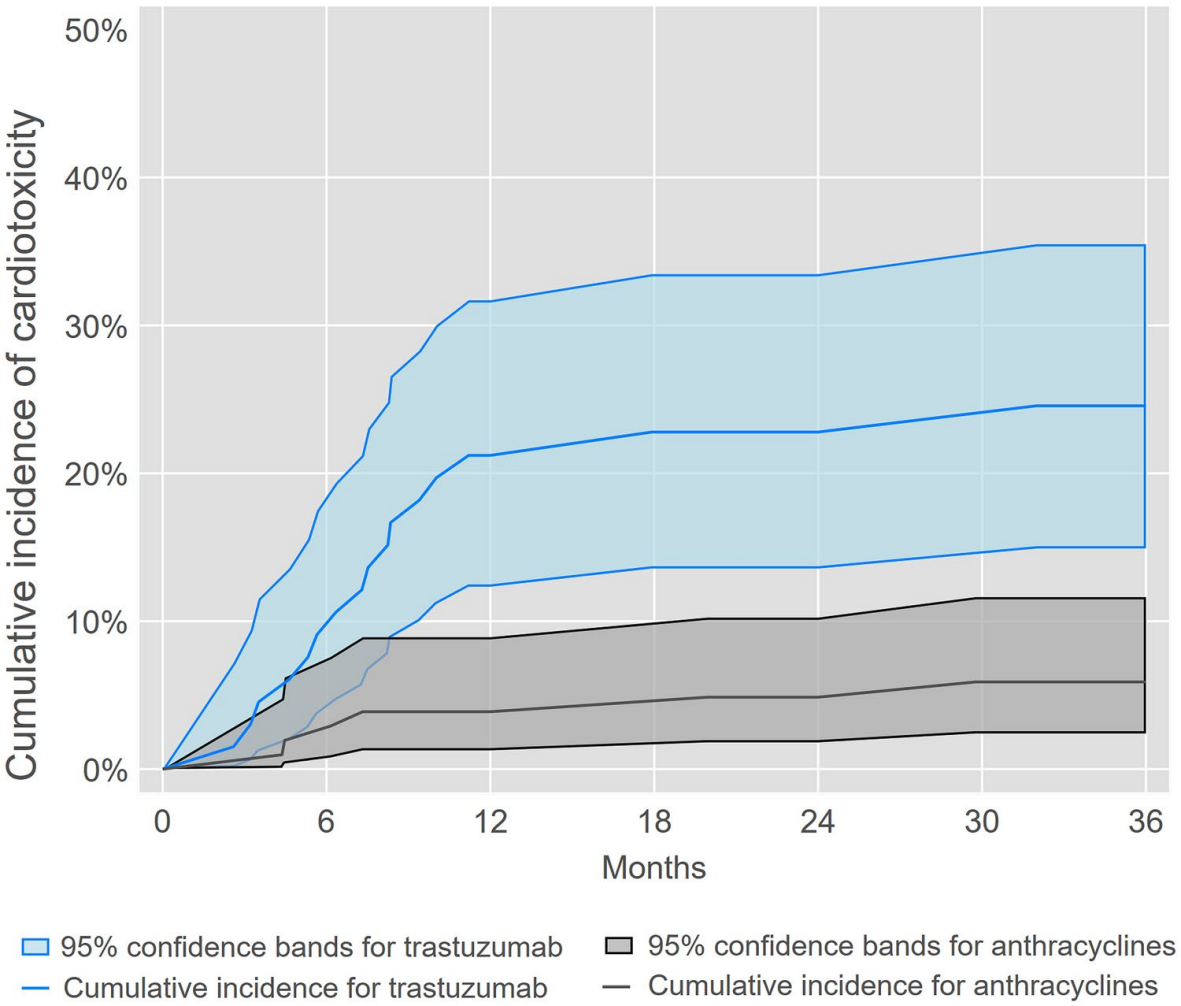
Cumulative incidence of cardiotoxicity^a^ among socioeconomically marginalized women diagnosed with first primary invasive breast cancer who initiated trastuzumab or anthracyclines. ^**a**^Cardiotoxicity was defined as heart failure or cardiomyopathy using diagnosis codes that were confirmed by supporting documentation in EHR, or cardiac dysfunction indicated by drop of LVEF ≥ 10% to < 55% after trastuzumab or anthracycline initiation

**Fig. 3:**
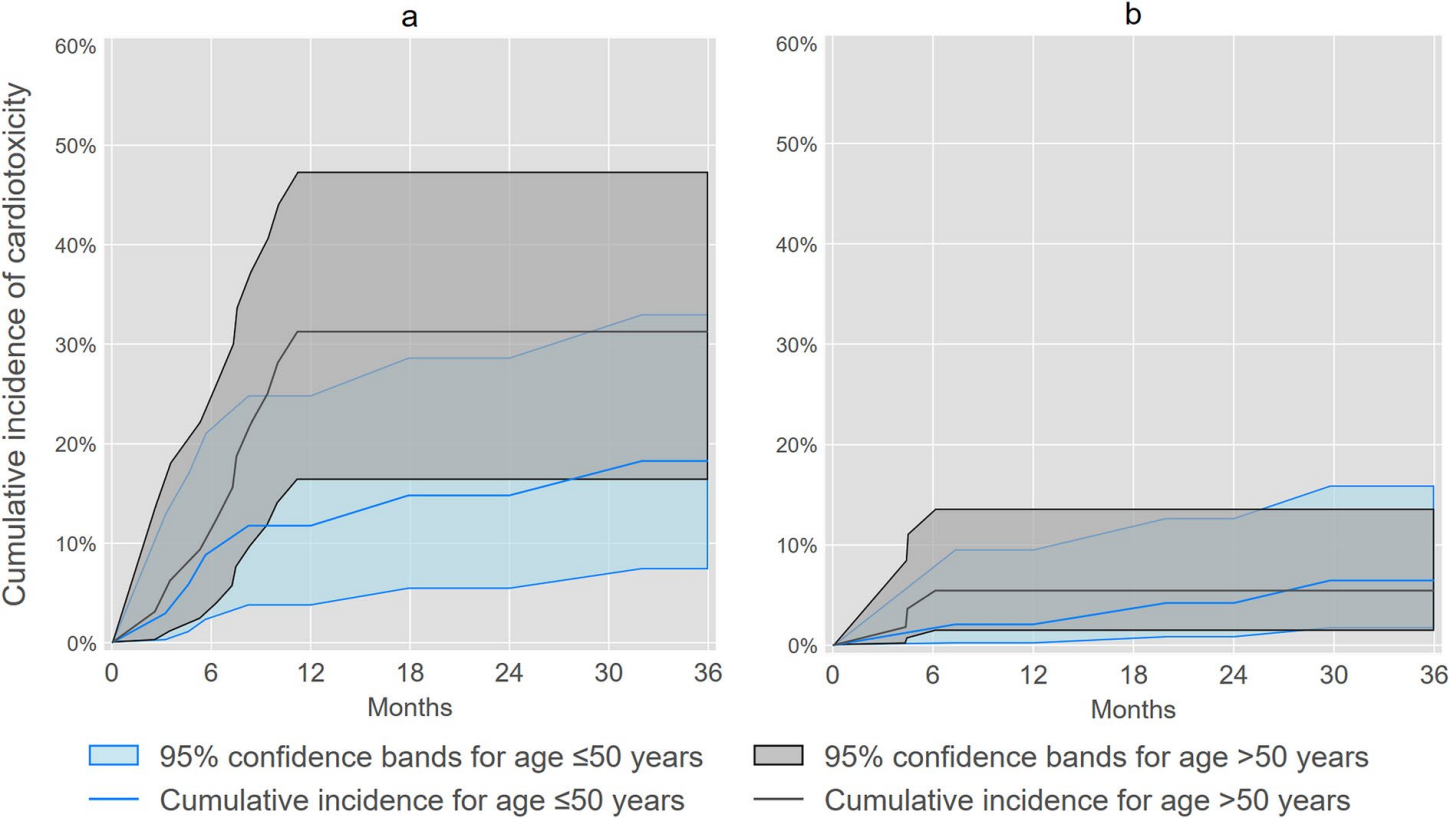
Age-group-specific cumulative incidence of cardiotoxicity^a^ among socioeconomically marginalized women diagnosed with first primary invasive breast cancer who initiated trastuzumab or anthracyclines. **a** Trastuzumab. **b** Anthracycline. ^**a**^Cardiotoxicity was defined as heart failure or cardiomyopathy using diagnosis codes that were confirmed by supporting documentation in EHR, or cardiac dysfunction indicated by drop of LVEF ≥ 10% to < 55% after trastuzumab or anthracycline initiation

We identified 22 incident cases of cardiotoxicity within 3 years of trastuzumab or anthracycline initiation, of which five cases met our cardiac dysfunction criteria without a clinical diagnosis of heart failure or cardiomyopathy. Figure 2, 3, and 4 illustrate and Table 2 summarizes overall and subgroup-specific cumulative incidence of cardiotoxicity following trastuzumab or anthracycline initiation. Cumulative incidence of cardiotoxicity was 21% (95% CL: 12%, 32%) at one year and 25% (95% CL: 15%, 35%) at three years among women who initiated trastuzumab, whereas cumulative incidence was 3.9% (95% CL: 1.3%, 8.9%) at one year and 5.9% (95% CL: 2.4%, 12%) at three years for women who initiated anthracyclines. For trastuzumab initiation, cumulative incidence of cardiotoxicity at three years was 31% (95% CL: 16%, 47%) for women aged > 50 years and 18% (95% CL: 7.4%, 33%) for women aged ≤ 50 years. For anthracycline initiation, cumulative incidence of cardiotoxicity at three years was 5.5% (95% CL: 1.4%, 14%) for women aged > 50 years and 6.5% (95% CL: 1.7%, 16%) for women aged ≤ 50 years. Lastly, for trastuzumab initiation, cumulative incidence of cardiotoxicity at three years was 28% (95% CL: 10%, 49%) for women with left-sided radiation therapy and 23% (95% CL: 12%, 36%) for women without left-sided radiation therapy. For anthracycline initiation, cumulative incidence of cardiotoxicity at three years was 8.4% (95% CL: 2.2%, 20%) and 4.5% (95% CL: 1.2%, 11%) for women with or without left-sided radiation therapy, respectively.

**Fig. 4:**
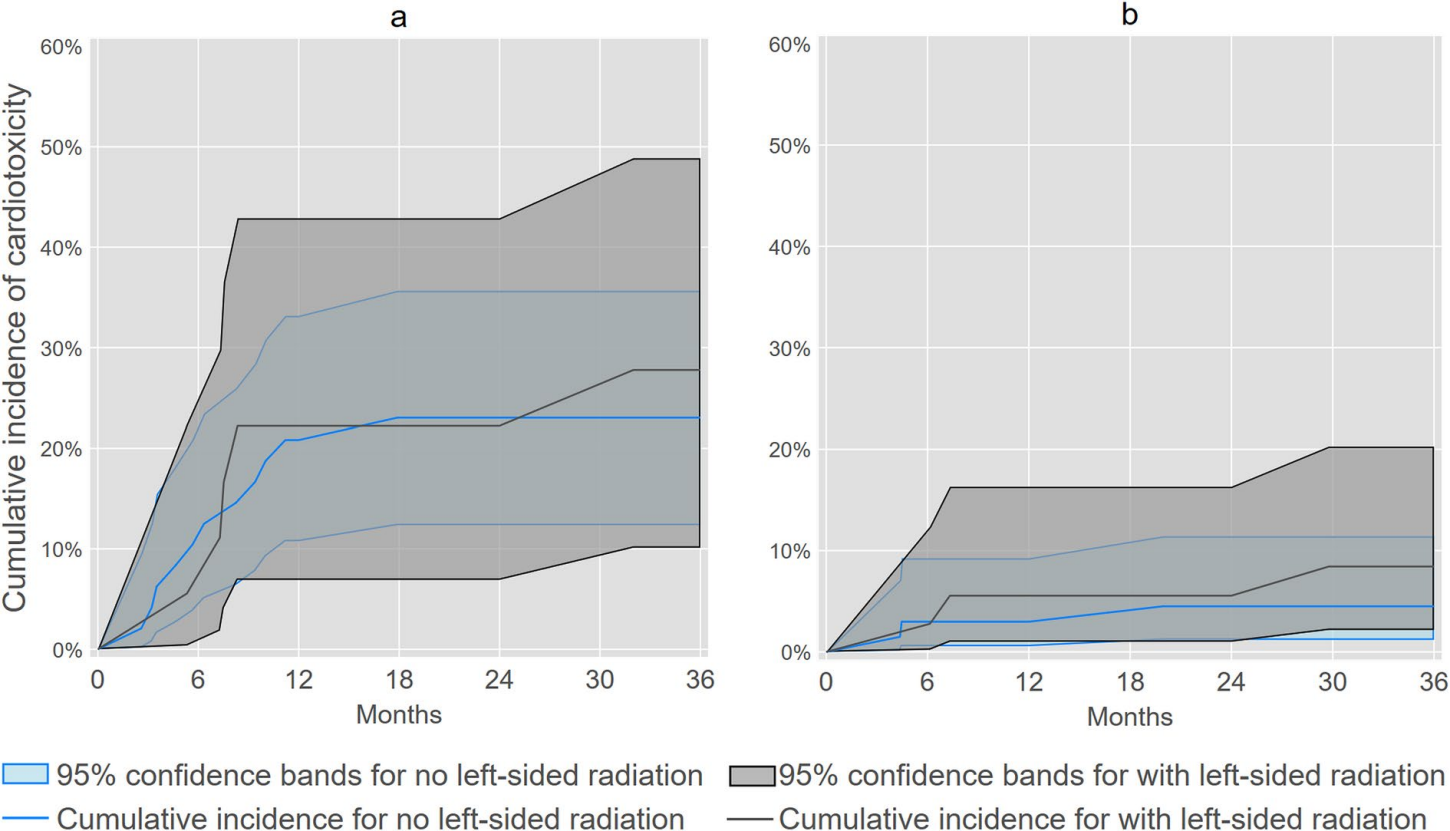
Cumulative incidence of cardiotoxicity^a^ among socioeconomically marginalized women diagnosed with first primary invasive breast cancer who initiated trastuzumab or anthracyclines by receipt of left-sided radiation. **a** Trastuzumab. **b** Anthracycline. ^**a**^Cardiotoxicity was defined as heart failure or cardiomyopathy using diagnosis codes that were confirmed by supporting documentation in EHR, or cardiac dysfunction indicated by drop of LVEF ≥ 10% to < 55% after trastuzumab or anthracycline initiation

**Table 2:** Overall and subgroup-specific cumulative incidence of cardiotoxicity among socioeconomically marginalized women diagnosed with first primary invasive breast cancer who initiated trastuzumab or anthracyclines

	1-year cumulative incidence (95% CL^b^)	2-year cumulative incidence (95% CL^b^)	3-year cumulative incidence (95% CL^b^)
**Overall cardiotoxicity**^a^			
Trastuzumab	21% (12%, 32%)	23% (14%, 33%)	25% (15%, 35%)
Anthracycline	3.9% (1.3%, 8.9%)	4.9% (1.8%, 10%)	5.9% (2.4%, 12%)
**Age-group specific cardiotoxicity**^a^			
*Age ≤ 50 years*			
Trastuzumab	12% (3.7%, 25%)	15% (5.4%, 29%)	18% (7.4%, 33%)
Anthracycline	2.1% (0.17%, 9.6%)	4.2% (0.77%, 13%)	6.5% (1.7%, 16%)
*Age > 50 years*			
Trastuzumab	32% (16%, 47%)	32% (16%, 47%)	32% (16%, 47%)
Anthracycline	5.5% (1.4%, 14%)	5.5% (1.4%, 14%)	5.5% (1.4%, 14%)
**Receipt of left-sided radiation therapy**			
*Yes*			
Trastuzumab	22% (6.9%, 43%)	22% (6.9%, 43%)	28% (10%, 49%)
Anthracycline	5.6% (1.0%, 16%)	5.6% (1.0%, 16%)	8.4% (2.2%, 20%)
*No*			
Trastuzumab	21% (11%, 33%)	23% (12%, 36%)	23% (12%, 36%)
Anthracycline	3.0% (0.56%, 9.3%)	4.5% (1.2%, 11%)	4.5% (1.2%, 11%)

^a^Cardiotoxicity was defined as heart failure or cardiomyopathy using diagnosis codes that were confirmed by supporting documentation in EHR, or cardiac dysfunction indicated by drop of LVEF ≥ 10% to < 55% after trastuzumab or anthracycline initiation

^**b**^Confidence limits

*Table **3* summarizes clinical consequences of cardiotoxicity. Interruption of trastuzumab or anthracycline therapy was experienced by 59% of patients with cardiotoxicity, of whom 38% were unable to continue cancer-directed treatment. Cardiotoxicity was managed using medications for 41% of patients, whether as new prescriptions or new doses of existing prescriptions for cardiovascular disease management. Prescribed medications included angiotensin-converting enzyme (ACE) inhibitors, angiotensin II receptor blockers (ARBs), or a combination of diuretics and beta-blockers. Cardiotoxicity following trastuzumab initiation resolved or partially resolved for 81% patients within one year, whereas cardiotoxicity did not resolve within one year for any patients who initiated anthracyclines.

**Table 3:** Clinical consequences of cardiotoxicity within 3 years of trastuzumab or anthracycline initiation among socioeconomically marginalized women diagnosed with first primary invasive breast cancer

	HER2-positive and initiated trastuzumab (*n* = 16) *n* (%)	HER2-negative and initiated anthracyclines (*n* = 6) *n* (%)
*Impact on trastuzumab or anthracycline therapy completion*		
Cardiotoxicity occurred after treatment completion	0 (0)	3 (50)
Completed treatment without interruption	3 (19)	2 (33)
Treatment interrupted but resumed and completed	8 (50)	0 (0)
Treatment interrupted and did not complete	4 (25)	1 (17)
Lost to follow-up	1 (6.3)	0 (0)
Cardiotoxicity management		
Treated with new medication or new doses of existing medication	5 (31)	4 (67)
Continued with medications for existing cardiovascular disease	4 (25)	0 (0)
Observation, no medication	7 (44)	2 (33)
*Cardiotoxicity outcome*		
Reversed or partially reversed^a^	13 (81)	0 (0)
Not reversed^b^	2 (13)	4 (67)
Not evaluated or lost to follow-up	1 (6.3)	2 (33)

^a^LVEF reversed to ≤ 5% of baseline, improved by ≥ 10% but remained > 5% below baseline, or had clinical documentation of improved symptoms for patients without follow-up LVEF measurement

^b^LVEF improved by < 10% and remained > 5% below baseline or had no clinical documentation of improved symptoms for patients without follow-up LVEF measurement

Our sensitivity analysis using a stricter definition of cardiac dysfunction (i.e., LVEF dro*p* > 15% to < 40%) resulted in lower estimates for cumulative incidence among women who initiated trastuzumab (1-year cumulative incidence: 11%, 95% CL: 4.7%, 19%; 3-year cumulative incidence: 15%, 95% CL: 7.9%, 25%), but cumulative incidence estimates did not change for women who initiated anthracyclines (Supplementary Materials, Appendix A, Supplementary Table S2 and Fig. S1).

## Discussion

We observed substantial overall risk of cardiotoxicity among socioeconomically marginalized breast cancer patients, particularly within the first year after initiation of either anthracyclines or trastuzumab. The observed pattern may be consistent with early cardiotoxicity. In addition, we observed modest differences in point estimates of cardiotoxicity risk by age group and left-sided radiation, but imprecision precludes definitive understanding of variation in cardiotoxicity risk for these subgroups. Lastly, more than half of patients with cardiotoxicity experienced interruption of trastuzumab or anthracycline administration, but unlike anthracyclines, the majority of cardiotoxicity cases following trastuzumab initiation resolved or partially resolved within one year.

### Limitations

Proper interpretation of our results requires consideration of potential biases. Cardiotoxicity may be misclassified if LVEF was not systematically assessed for all patients during follow-up. The magnitude of misclassification may be mitigated by our use of either LVEF decline or diagnostic codes for heart failure and cardiomyopathy. Nevertheless, systematic assessment of LVEF would have allowed greater sensitivity for detecting cardiotoxicity. The potential consequence of misclassification bias is underestimated cardiotoxicity risk.

Our analysis assumes noninformative censoring (i.e., patients were censored at random). This assumption may be violated (i.e., informative censoring) in studies of clinical cohorts based on routinely collected data [23, 29], such as ours. Informative censoring is a form of selection bias, where individuals who were censored are systematically different with respect to the outcome from individuals who remained in the cohort. For example, individuals were censored in our study 12 months after the last encounter with the health system. If individuals who were lost to follow-up had higher risk of cardiotoxicity than individuals who remained in the cohort, then cardiotoxicity risk may be underestimated in our study. Nevertheless, only 8% of our patients were censored because of loss to follow-up, which mitigates potential underestimation.

Our socioeconomically marginalized population has barriers to care, which raises questions about potential selection bias because of challenges distinguishing between prevalent and incident heart failure or cardiomyopathy. For example, 57% of cancer patients (51% of women with breast cancer) in our health system did not have a documented primary care visit within 2 years prior to cancer diagnosis (Supplementary Materials, Appendix B). If heart failure or cardiomyopathy were prevalent but undiagnosed, then these cases would have been ineligible for our study. The potential consequence of this selection bias is overestimated cardiotoxicity risk, but the effect of this bias may be inconsequential considering that prevalent cases would have been detected for the 90% of patients who had LVEF measurements prior to trastuzumab or anthracycline initiation. Therefore, the net effect of these misclassification and selection biases may be underestimated cardiotoxicity risk in our study.

### Cumulative evidence

Several meta-analyses reported cardiotoxicity risk following trastuzumab or anthracycline exposure [7, 8, 30–35], but these meta-analyses summarized risk estimates from studies with substantial clinical and methodologic heterogeneity. For example, estimates were summarized across studies despite differences in geographic locations, cardiotoxicity definition (e.g., LVEF thresholds of 55%, 53%, or 40%), cancer types (e.g., breast, lymphoma, lung), treatments (e.g., anthracycline or trastuzumab alone or in combination), study design (e.g., randomized controlled trials or observational studies using routinely collected data), duration of follow-up, and methods of risk estimation (e.g., incidence proportions without a common duration of follow-up or cumulative incidence with or without competing events). Studies with clinical and methodologic heterogeneity are incomparable, and summary estimates in this scenario are misleading [36–39].

To facilitate comparability with our study, we identified prior studies included in meta-analyses[7, 8, 30–35] that: (1) were observational studies using routinely collected data, (2) estimated cardiotoxicity risk following trastuzumab or anthracycline exposure among women with breast cancer in the United States, and (3) used cardiotoxicity definitions similar to our study. We identified six relevant studies [40–45], of which three studies reported cardiotoxicity risks of 1.0% – 3.8% after 1 year and 7.8 – 11% after 3 years following trastuzumab exposure.[40, 44, 45] These estimates were substantially lower than our estimates for trastuzumab (1-year: 22%, 95% CL: 13%, 32%; 3-year: 25%, 95% CL: 15%, 36%) even compared with estimates from our sensitivity analysis that used a stricter definition of cardiotoxicity (1-year: 11%, 95% CL: 4.8%, 20%; 3-year: 16%, 95% CL: 8.2%, 26%). Bowles et al. [40] reported lower (2.7%), while Du et al. [41] reported higher (8.3% for congestive heart failure and < 5% for cardiomyopathy) anthracycline-induced cardiotoxicity than our study at three years. Nevertheless, the median age was 70 years for the study population of Du et al. [41], which is 20 years more than the median age of our population. Older age is strongly associated with cardiotoxicity [2, 46, 47], which may explain the difference in cardiotoxicity risk between our study and the study by Du et al.[41] Two studies [42, 43] reported notably higher cardiotoxicity than our study (32 – 33% with approximately 1-year follow-up), which may be explained by the inclusion of patients treated with both trastuzumab and anthracyclines. Several prior studies [40, 41, 45] also overestimated cardiotoxicity risk because of the method used for risk estimation. Specifically, cumulative incidence of cardiotoxicity was estimated using the complement of the Kaplan–Meier estimate. This approach consistently overestimates risk because death during follow-up is censored rather than accounted as a competing event [21, 48] Some studies reported cardiotoxicity incidence proportion[42–44] which ignores duration of follow-up and timing of cardiotoxicity incidence.

Our study population had a higher proportion of racial/ethnic minorities than prior studies (e.g., 34% vs. 5.6 – 10% for non-Hispanic Black, and 30% vs. 3.2% for Hispanic) [40, 41, 45]. Racial/ethnic minorities have a high prevalence of risk factors for cardiac dysfunction such as obesity and tobacco use [49, 50]. We speculate that high cardiotoxicity risk observed in our study may be partially attributable to high prevalence of such risk factors at cancer diagnosis [2]. For example, the prevalence of obesity in our socioeconomically marginalized population was 61%, which exceeds the 37 – 49% obesity reported in previous breast cancer studies with limited socioeconomic diversity [51, 52]. Current tobacco use was 21% at the time of cancer diagnosis in our population, which is higher than current use at diagnosis reported in prior studies (6 – 20%) [43, 53–55]. Hypertension prevalence was 46% in our population, whereas hypertension prevalence in prior cardiotoxicity studies among breast cancer patients was 28 – 32% [43, 44, 55]. Lastly, diabetes prevalence was 24% in our population, whereas diabetes prevalence in prior studies was 1 – 11% [43, 44, 55]. Management of these conditions and behaviors are critical for general cardiovascular health, but more evidence is needed about whether the effects would extend to decreasing cardiotoxicity risk related to cancer-directed therapy [56].

### Implications

Our findings address a gap in the evidence about cardiotoxicity among socioeconomically marginalized breast cancer patients. The high risk of cardiotoxicity following trastuzumab observed in our study raises questions about whether shorter duration (e.g., 6 months) may be a reasonable option to reduce potential cardiotoxicity for socioeconomically marginalized breast cancer patients. Evidence regarding the effectiveness of shorter duration trastuzumab therapy is inconsistent but generally indicates reduced effectiveness and may not justify reduced harms [57–59]. Nevertheless, this evidence is based on trials with inadequate representation of socioeconomically marginalized breast cancer patients, which compromises generalizability and reiterates the need for greater diversity in clinical trials [60]. Meanwhile, evidence from our study may be useful in settings that provide care for socioeconomically marginalized breast cancer patients, particularly for raising awareness about potential unmet needs for cardiotoxicity prevention, detection, and management. Care coordination between cardiologists and oncologists may be useful for personalizing cardiotoxicity prevention, detection, and management. Such care decisions may be further supported by clinical prediction models for risk stratification [61, 62]. Treatment modifications, such as anthracycline dose reduction, administration schedule modifications, and analogous treatment options may need to be considered for achieving optimal treatment effectiveness while minimizing cardiotoxic effects [18, 63]. Ultimately, optimal strategies for preventing, detecting, and managing cardiotoxicity will need to address prior and ongoing consequences of barriers to care and unmet needs of socioeconomically marginalized breast cancer patients.

## Supplementary Information

Below is the link to the electronic supplementary material.

Supplementary file1 (PDF 294 KB)

## Data Availability

The data analyzed for the current study are available on reasonable request to the corresponding author and review by the JPS Health Network External Data Governance Committee (research@jpshealth.org).
