# Supplementary material for: Cardiotoxicity among socioeconomically marginalized breast cancer patients
Source: Breast Cancer Res Treat. 2022 Aug 15;195(3):401–11. doi: 10.1007/s10549-022-06695-0 (PMC9464741; doi:10.1007/s10549-022-06695-0)
Supplement: Supplementary file 1 — Supplementary file1 (PDF 294 KB) [file 10549_2022_6695_MOESM1_ESM.pdf]

Breast Cancer Research and Treatment

Cardiotoxicity among socioeconomically marginalized breast cancer patients

Yan Lu, Aaron W. Gehr, Ifedioranma Anikpo, Rachel J. Meadows, Kevin J. Craten,
Kalyani Narra, Anuradha Lingam, Sandeep Kamath, Bhavna Tanna, Bassam Ghabach,
Rohit P. Ojha

Corresponding author:
Rohit P. Ojha, DrPH, FACE
Center for Epidemiology and Healthcare Delivery Research
JPS Health Network
1500 South Main Street
Fort Worth, TX 76104
Email: rojha@jpshealth.org
Phone: 817-702-1298

SUPPLEMENTARY MATERIALS

Appendix A

Supplementary Table S1 International Classification of Diseases, 9th (ICD-9) or 10th Edition (ICD-10) diagnosis codes used for heart failure or cardiomyopathy

	ICD-9	ICD-10
**Heart failure**	428.xx	I50.xx
**Cardiomyopathy**	425.0, 425.1, 425.2, 425.3, 425.4, 425.7, 425.8, 425.9, 425.11, 425.18	I42.0, I42.1, I42.2, I42.3, I42.4, I42.5, I42.7, I42.8, I42.9

Supplementary Table S2 Distribution of cardiovascular disease risk factors among socioeconomically marginalized women diagnosed with first primary invasive breast cancer by cardiotoxicity status within 3 years of trastuzumab or anthracycline initiation

	HER2 positive and initiated trastuzumab		HER2 negative and initiated anthracyclines	
	**No cardiotoxicity within 3 years (*n*=50) *n* (%)**	**Cardiotoxicity within 3 years (*n*=16) *n* (%)**	**No cardiotoxicity within 3 years (*n*=97) *n* (%)**	**Cardiotoxicity within 3 years (*n*=6) *n* (%)**
**BMI**				
**BMI<25**	7 (14)	5 (31)	8 (8.3)	1 (17)
**25≤BMI<30**	11 (22)	1 (6.3)	29 (30)	2 (33)
**BMI≥30**	32 (64)	10 (63)	59 (61)	3 (50)
**Missing**	0 (0)	0 (0)	1 (1.0)	0 (0)
**Tobacco use**				
**Never used**	33 (66)	9 (56)	59 (61)	3 (50)
**Current user**	8 (16)	3 (19)	23 (24)	2 (33)
**Former user**	9 (18)	4 (25)	14 (14)	1 (17)
**Missing**	0 (0)	0 (0)	1 (1.0)	0 (0)
**Alcohol use**				
**Never used**	43 (86)	13 (81)	81 (84)	6 (100)
**Current user**	6 (12)	3 (19)	13 (13)	0 (0)
**Former user**	0 (0)	0 (0)	2 (2.1)	0 (0)
**Missing**	1 (2.0)	0 (0)	1 (1.0)	0 (0)
**Hypertension at baseline**				
**Yes**	21 (42)	11 (69)	46 (47)	0 (0)
**No**	29 (58)	5 (31)	51 (53)	6 (100)
**Diabetes at baseline**				
**Yes**	8 (16)	7 (44)	26 (27)	0 (0)
**No**	42 (84)	9 (56)	71 (73)	6 (100)

Supplementary Table S3 Cumulative incidence of cardiotoxicity[a] for human epidermal growth factor receptor 2 (HER2) positive patients initiated trastuzumab and HER2 negative patients initiated anthracyclines among socioeconomically marginalized women diagnosed with first primary invasive breast cancer

	1-year cumulative incidence (95% CL[b])	2-year cumulative incidence (95% CL[b])	3-year cumulative incidence (95% CL[b])
**Overall cardiotoxicity[a]**			
**Trastuzumab**	11% (4.7%, 19%)	14% (6.7%, 23%)	15% (7.9%, 25%)
**Anthracycline**	3.9% (1.3%, 8.9%)	4.9% (1.8%, 10%)	5.9% (2.4%, 12%)

[a]Cardiotoxicity was defined as heart failure or cardiomyopathy using diagnosis codes that were confirmed by supporting documentation in EHR, or drop of LVEF >15% to <40% after trastuzumab or anthracycline initiation
[b]Confidence limits

Supplementary Fig. S1 Cumulative incidence of cardiotoxicity[a] curves for human epidermal growth factor receptor 2 (HER2) positive patients who initiated trastuzumab and HER2 negative patients who initiated anthracyclines among socioeconomically marginalized women diagnosed with first primary invasive breast cancer

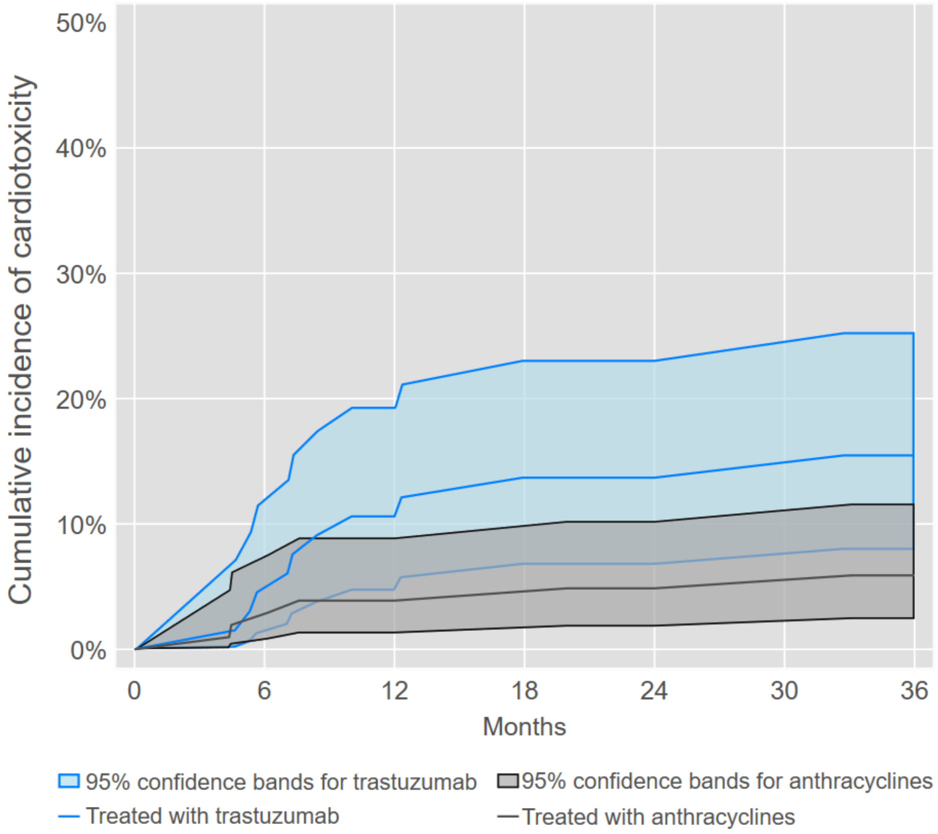

[a] Cardiotoxicity was defined as heart failure or cardiomyopathy using diagnosis codes that were confirmed by supporting documentation in EHR, or drop of LVEF >15% to <40% after trastuzumab or anthracycline initiation

Appendix B

Assessment of primary care use among socioeconomically marginalized cancer patients prior to cancer diagnosis

We linked institutional oncology registry data from JPS Oncology and Infusion Center with JPS Health Network electronic health records (EHR) to assess primary care use prior to cancer diagnosis. Our eligible population included patients aged ≥18 years diagnosed with first primary cancer in 2017. The outcome of interest was primary care visit at JPS Health Network, which was defined as at least one completed encounter at any of the 14 primary care clinics of JPS Health Network within two years prior to cancer diagnosis. We estimated overall and breast cancer-specific proportions with 95% confidence limits (CL) of no primary care visit two years prior to cancer diagnosis.

Overall, 57% (95% CL: 54%, 60%) of cancer patients had no primary care visit two years prior to cancer diagnosis. Among women diagnosed with breast cancer, 51% (95% CL: 43%, 58%) had no primary care visit two years prior to cancer diagnosis. These estimates are specific to primary care use at JPS Health Network and do not account for possible primary care use at other health systems.
